# Chitinases from Bacteria to Human: Properties, Applications, and Future Perspectives

**DOI:** 10.1155/2015/791907

**Published:** 2015-11-19

**Authors:** Abhishek Singh Rathore, Rinkoo D. Gupta

**Affiliations:** Faculty of Life Sciences and Biotechnology, South Asian University, New Delhi 110021, India

## Abstract

Chitin is the second most plenteous polysaccharide in nature after cellulose, present in cell walls of several fungi, exoskeletons of insects, and crustacean shells. Chitin does not accumulate in the environment due to presence of bacterial chitinases, despite its abundance. These enzymes are able to degrade chitin present in the cell walls of fungi as well as the exoskeletons of insect. They have shown being the potential agents for biological control of the plant diseases caused by various pathogenic fungi and insect pests and thus can be used as an alternative to chemical pesticides. There has been steady increase in demand of chitin derivatives, obtained by action of chitinases on chitin polymer for various industrial, clinical, and pharmaceutical purposes. Hence, this review focuses on properties and applications of chitinases starting from bacteria, followed by fungi, insects, plants, and vertebrates. Designing of chitinase by applying directed laboratory evolution and rational approaches for improved catalytic activity for cost-effective field applications has also been explored.

## 1. Introduction

Chitin is a polymer of N-acetylglucosamine, linked with *β*-1,4-glycosidic bonds. It is a major portion of cell walls of fungi, exoskeleton of insects, and crustacean shells. Despite its abundance, chitin does not accumulate in the environment due to presence of chitinolytic enzymes known as “chitinases.” Several organisms including bacteria, fungi, insects, plants, and animals produce chitinases. Detailed structural and mechanical differences among the chitinases produced by these organisms are reviewed by Adrangi and Faramarzi [1]. These organisms produce numerous types of chitinolytic enzymes. Many bacteria including* Serratia* and* Bacillus* are known to produce four different types of chitinases. However, most of the filamentous fungi are known to produce up to 20 different chitinases [2]. They act together in synergistic manner to catabolize chitin. These enzymes are able to degrade the chitin present in the cell walls of fungi as well as the exoskeletons of insects. They have shown being the potential agents for biological control of the plant diseases caused by various pathogenic fungi and insect pests that can be used as an alternative to chemical pesticides. There has been steady increase in demand of chitin derivatives, obtained by action of chitinases on chitin polymer for various industrial, clinical, and pharmaceutical purposes. However, there are immense challenges for the production of specific and cost-effective enzymes. Possibilities of several potential applications of chitinases make it an interesting target enzyme for protein engineering. Considering the significant contribution of chitinases as biopesticides and in various industrial and pharmaceutical purposes, we aim to articulate properties of chitinases starting from bacteria, which is followed by fungi, insects, plants, and animals. Comprehensive applications of the chitinases are also explored. At the end, we aim to survey the future perspectives of chitinases by applying protein-engineering techniques.

## 2. Structures and Functions of Chitin

Chitin is a linear stable polymer of *β*-1,4-N-acetylglucosamine (GlcNAc), which is 2-acetamido-2-deoxy-D-glucose, and is the second highest occurring biopolymer on the planet after cellulose [3]. It is present in exoskeleton of insect, crabs, shrimp, lobsters, fungi, yeast, diatoms, nematodes, crustaceans, and other invertebrates. Percentage of chitin found varies with different organisms. Highest percentage of chitin is found in crabs and shrimps, which makes 90% of chitinous waste. It is a crystalline polysaccharide that exists in nature in three different forms: *α*-chitin, *β*-chitin, and *ϒ*-chitin. *α*-Chitin is the most abundant, isomorphic, and more compact form due to the arrangement of chitin chains in antiparallel fashion which favors strong hydrogen bonding. *β*-Chitin is loosely packed as in this chitin, and chains are arranged in parallel fashion with weaker intermolecular forces which leads to less stable form. Third polymorphic form is *ϒ*-chitin which is a mixture of both *α*- and *β*-chitins [4]. Hydrolysis of glycosidic bonds of chitin is catalyzed by action of chitinases which degrades chitin into disaccharides and longer oligosaccharides. *β*-chitin is easily hydrolyzed and more soluble as compared to *α*-chitin. It can be swollen in water as well as dissolved in formic acid [5].

Chitin mainly helps to form exoskeleton as in arthropods and some species of fungi; in addition chitin in several species is associated with many proteins, which determine whether the exoskeleton will be rigid, soft, or flexible. It also associates with non-protein part such as calcium carbonate that forms the structure in shrimp, crab, and lobsters. Chitin and its related materials have extensive applications in drug delivery: wound healing, dietary fiber, and wastewater treatment [6]. Also, chitin provides protective benefits by forming exoskeleton in arthropods and fungi.

## 3. Classifications and Catalytic Mechanism of Chitinases

**Figure 1:**
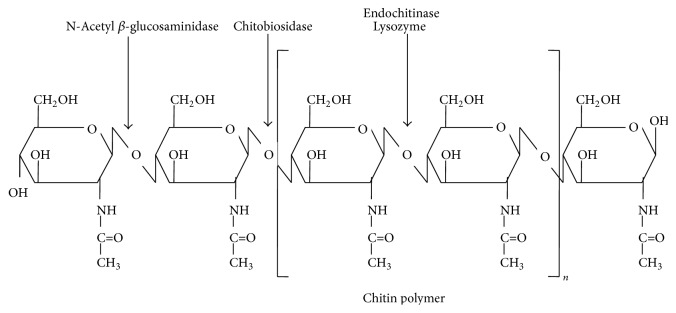
Specificity of different chitinases towards chitin polymer. N-Acetyl *β*-glucosaminidase cleaves monomeric unit of GlcNAc from nonreducing terminal. Chitobiosidase cleaves dimeric unit of GlcNAc from nonreducing terminal. Endochitinase cleaves glycosidic bond randomly at internal sites in chitin polymer.

Chitinases (EC 3.2.2.14) are glycosyl hydrolases, characterized for hydrolyzing *β*-1,4 linkage of N-acetyl glucosamine present in chitin chains which vary between the size ranging from 20 kDa to about 90 kDa [7]. In natural state, chitin is tightly bound with lipid pigments, proteins, and minerals like calcium carbonate; hence preparation of chitin includes deproteinization and demineralization of chitinous waste by strong acid or a base. These processes involve high cost, low yield, and corrosion problems due to which cost of its oligomers is much. Therefore, chitinolytic enzymes are important component in utilization of chitinous waste and solve environmental problems as they are biodegradable and inexpensive. Chitinolytic enzymes have been divided into two main categories: endochitinases (EC 3.2.1.14) and exochitinases. Endochitinases are the class of chitinases that cleaves randomly at internal sites in polymer of chitin generating low molecular mass multimers of glucosamine residues such as chitotriose, chitobiose, and diacetylchitobiose (Figure 1). Exochitinases have been classified into two categories, namely, chitobiosidases (EC 3.2.1.29) which catalyze progressive release of diacetylchitobiose from terminal nonreducing end and N-acetylglucosaminidases (EC 3.2.1.30) which cleaves oligomeric products obtained by endochitinases into monomers of N-acetyl glucosamine (GlcNAc) [8].

**Figure 2:**
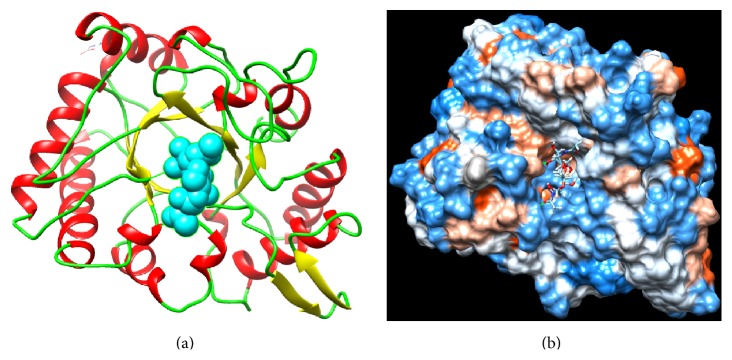
Interaction of GlcNAc dimer TIM-barrel (*β*/*α*)8 catalytic domain of family 18 bacterial endochitinase of* S*.* marcescens*: (a) Bacterial endochitinase interaction with GlcNAc dimer (blue); (b) Hydrophobic and hydrophilic regions around active site of chitinase C.

Based on amino acid similarity of chitinases from various organisms, they have been divided into five classes and have been categorized into families 18, 19, and 20 of glycosyl hydrolases [11]. Family 18 comprises chitinases from viruses, bacteria, fungi, animals, and certain plant chitinases. It consists of a number of conserved repeats of amino acids and enzyme core, which has 8 strands of parallel *β* sheets, creating a barrel positioned down *α* helices, in turn forming a ring towards the outside (Figure 2) [12]. Family 19 comprises some plant chitinases and* Streptomyces* chitinases [13]. Families 18 and 19 do not share amino acid sequence similarity, and they have completely different 3D structure and therefore have been said to evolve from different ancestors. Family 20 involves N-acetylglucosaminidases from bacteria, certain fungi, and humans.

## 4. Role of Chitinases in Various Organisms

Mainly, organisms require these chitinolytic enzymes for three different purposes. (a) organisms possess a tough layer of chitin and chitinases are expressed during developmental phases to help in the remodeling of their exoskeleton so as to maintain and support body size and shape. (b) Organisms that consume other chitin containing organisms as a source of nutrient express chitinases to digest the insoluble chitin polymer into absorbable metabolites, which gives energy. (c) Organisms that are prone to infection by chitin-coated microorganisms express chitinases to degrade the protective shield of the infecting pathogens, thereby providing immunity. Different forms and specific functions of chitinases in bacteria, fungi, insects, plants, and vertebrates are described below for comprehensive appraisal.

### 4.1. Bacteria

Bacteria mainly produce chitinases in order to supply nitrogen and carbon as a source of nutrients or precursors and parasitism [14, 15]. They are used for degradation of chitin and its utilization as an energy source [16]. Chitinases play an important role in bacterial pathogenesis wherever host contains chitin [17].* Serratia marcescens*, one of the best studied chitinolytic bacteria, has been reported producing mainly four types of chitinases ChiA, ChiB, ChiC, and CBP21 (chitin binding protein). All three chitinases belong to family 18 of glycosyl hydrolases with (*β*/*α*) 8 TIM-barrel catalytic domain with approximately six sugar subsites [18]. ChiA and ChiB have multimodular organization, that is, have an N-terminal chitin binding module with a fibronectin-like fold in ChiA or a C-terminal CBM5 module. CBM modules found in chitinases are distantly related, and they are characterized by presence of conserved exposed tryptophan residues that interact with the substrate [19]. Presence of this domain increases the substrate binding affinity as well as efficiency of chitin hydrolysis, particularly for more crystalline forms of chitin [20].

Marine bacteria such as* Vibrio* are well studied because they solely live on chitin, which is highly abundant in marine ecosystems. Therefore they serve as an ideal candidate for bioconversion of chitin biomaterials for various purposes. The chitin degradation machinery of* Vibrio* is proved to be highly efficient. Structure and functions of chitinases and chitin uptake system in* Vibrio* are well studied. Songsiritthigul has described that the interacting sugars undergo conformational changes prior to hydrolysis by the wild-type enzyme with the help of crystallographic data obtained by four X-ray structures of* Vibrio harveyi* chitinase A and its catalytically inactive mutant (E315M) in the presence and absence of substrates [21]. Recently, chitoporin (VhChiP), a sugar-specific channel responsible for the transport of chitooligosaccharides through the outer membrane of the marine bacterium* Vibrio harveyi*, has been reported by Suginta and group [22]. VhChiP is found to be the most potent sugar-specific channel reported to date, with its high efficiency. This reflects apparently the need for the bacterium to take up chitin containing nutrients in aquatic conditions as its sole source of energy. Previous reviews, intensive on bacterial chitinases only, have signified the structural and functional aspects in great detail [1, 7, 14].

### 4.2. Fungi

Fungal chitinases play important role in nutrition, morphogenesis, and developmental process and are known to be produced at various stages during fungus growth [23]. Fungal chitinases belong to GH18 family of glycoside hydrolases which showed little amino acid sequence similarity with class 3 plant chitinases [8]. The fungal chitinases are divided into three groups, being chitinases A, B, and C on the basis of sequence and structural similarities [24]. Fungal chitinase A is processive chitinase with a singular catalytic domain having deep substrate binding site and no CBMs. Type B is nonprocessive chitinases and they have a CBM or a serine/threonine-rich domain on C-terminal of their catalytic domain. Type C fungal chitinases are also processive in nature due to their deep substrate binding site. They have a CBM on the N-terminal of catalytic domain. A special feature of fungal chitinase C is that it comprises several lysine motifs (LysM) also known as CBM 50 [2]. LysM containing receptors in plants play an important role in formation of root nodules in leguminous plants by binding to Nod factor [25]. Fungal chitinase C is found to expressed in many mycoparasitic fungi. A study on mycoparasitic* Trichoderma* species showed that these enzymes are involved in degradation of both self and non-self-cell walls [26]. Protection of self-cell wall in fungi is achieved by restricting the access to chitin by cell surface proteins.

Entomopathogenic fungi can play a major role as a biocontrol agent to control insects and pests. Overexpression and engineering of chitinases in these fungi increased their efficiency as a biocontrol agent [27]. Nematode parasitic fungi producing chitinase are used as biocontrol agent by targeting eggs and larva of plant and animal infecting nematodes [28]. Chitinase and chitin binding proteins can also be used for detection of invasive fungal infection in immunosuppressed patients [29].

The chitinases, which are associated with fungal cell wall, have role in filamentous fungal sporulation. This has been shown by using chitinase inhibitors demethylallosamidin and allosamidin, causing inhibition of fragmentation of hyphae into arthroconidia.* Trichoderma* have been considered as biocontrol agents in case of soil borne fungal pathogens amongst various chitinolytic fungi. Chitinases and *β*-1,3-glucanases from* Talaromyces flavus* and* Trichoderma* sp. have been purified and characterized. Hartl and group have given detailed insight into the diversity, mechanistic properties, and biotechnological potential of several other types of fungal chitinases [2].

### 4.3. Insects

All insect chitinases belong to GH18 family. On the basis of sequence similarity insect chitinases are divided into eight groups, which vary in their molecular weight ranging between 40 kDa and 85 kDa and their pH optima [30]. The overall structure consists of three domains: the catalytic region, a region enriched in the amino acids proline, glutamate, serine and threonine, and a cysteine-rich region. However, the last two domains are not necessary for chitinase activity. Cysteine-rich C-terminal chitin binding domain of insect chitinases anchored the substrate [2, 30].

Insect chitinases are involved in degradation of cuticle layer to smaller oligosaccharides by hydrolyzing and randomly breaking cuticle that is used for synthesis of new cuticle. In this way, these chitinases are required for partial degradation of old cuticle and synthesis of the new one. Expression of these enzymes is required to be under stringent control to avoid any premature exposure, which may lead to growth inhibition [31]. To achieve this goal insect produces specific chitinases that are differentially expressed over the course of time in different stages of metamorphosis [32]. Chitinases are also reported to be present in venom and salivary gland of some insects. In these cases, role of chitinases may be degradation of host cuticle.

### 4.4. Plants

Based on their amino acid sequence plant chitinases have been divided into 7 classes each possessing different structure [33]. Classes III and V of plant chitinase belong to GH18 family while the rest of the five classes belong to GH19 family [34]. The plant chitinases are generally endochitinases and smaller in molecular weight as compared to the insect chitinases. Many plants continuously express chitinases in tuber, stem, and root. In plants they act as pathogenesis related proteins, that is, synthesized in response to self-defense against phytopathogenic attack also at time these chitinases have been reported to take part in vital physiological processes of plants like embryogenesis and ethylene formation [12]. Chitinase is known to play a major role in defense against chitin containing pathogens like fungi and insects. Intensive research is being conducted to develop transgenic plants overexpressing various combinations of chitinases as well as other pathogenesis related proteins [35].

Chitinases are also known to be expressed during high environmental stress conditions such as cold and drought [8]. Some of the plant chitinases are found to show ice structuring tendency, which means they bind to ice crystals reducing their growth thus helping cold stress condition [36]. Some of class III chitinases are reported to play an important role in calcium storage without having more significant effect on their catalytic activity. The structures and catalytic functions of plant chitinases and their role in plant physiology have remarkably been reviewed by Sahai and Manocha [8] and Grover [37].

### 4.5. Vertebrates

In vertebrates, chitinases have been known to be the part of digestive tracts; for example, tGCase functions as a chitinolytic enzyme in the toad stomach [38] and acidic mammalian chitinases (AMCase) in the mouse gastrointestinal tract [39]. Chitotriosidase in human macrophages has been found in higher levels in lysosomal storage diseases as well as in patients with* Plasmodium falciparum* malarial infection [40] and chitin binding protein b04 (CBPb04) in bovine serum [41]. Since chitin is a main constituent of fungus cell wall and also present in mammalian diet, so role of mammalian chitinases is to provide innate immunity and also to digest the food. Human also produces AMCase; a family 18 chitinase is induced during TH2 inflammation through an interleukin dependent mechanism. Cytokines which appear to be of particular importance in asthma are lymphokines secreted by T-lymphocytes, IL-3 necessary for survival of mast cells and IL-5 important for growth, differentiation, and survival of eosinophils. Therefore, mammalian chitinases can be used as a biomarkers for asthma and hence inhibition of AMCase with chitinase inhibitors such as allosamidin reduces inflammation and can be used as potential target for asthma therapy [42]. Recently published research articles and reviews on human chitinases have implied its role in pathogenesis and as a biomarker [1, 43, 44].

## 5. Applications of Chitinases

**Figure 3:**
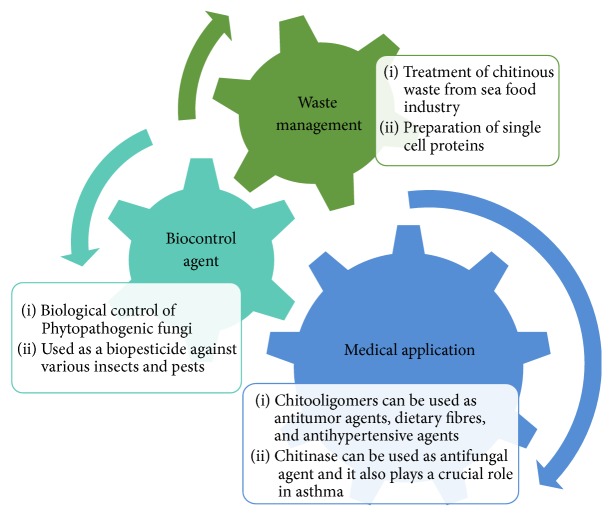
Industrial applications of chitinolytic enzymes.

Chitinases have several field applications. Chitinases are attaining prominence in the field of biotechnology applied in waste management, pest control in agriculture, and human health care which have been recapitulated in Figure 3 and discussed below in detail.

### 5.1. Waste Management

Recombinant chitinases can be used to convert chitinous biomass, that is, chitinous waste of marine organisms into simpler useful depolymerized components, hence reducing water pollution. Chitooligomers obtained by action of chitinases have a wide range of biotechnological applications in biochemical, food, and various chemical industries. Chitinase can also be used in conversion of chitinous waste into biofertilizers [45].

Another approach to utilize the chitinous waste effectively is production of single cell protein (SCP) [46]. In this approach chitinase degraded chitinous waste is used as carbon or nutritional source for production of biomass. Chitinase producing bacteria and yeast (e.g.,* S*.* marcescens* and* Pichia kudriavzevii*) can be used in aqua cultures for SCP production.

### 5.2. Biocontrol Agents

Chitinases are present in plants alongside various pathogenesis related proteins as a part of plant defense mechanism. So overexpression of a combination of various chitinases in transgenic plant may aid against fungal pathogens [35]. Chitinase can also be directly used as biopesticides against various fungi and insects that can be an alternative to chemical pesticides [47]. Other than being used directly as a biocontrol agent, chitinase can act as a target for biopesticide as chitin has a major role in insect metamorphosis as well as in gut of insects. A pseudo-trisaccharide allosamidin is an inhibitor of chitinase enzyme and it can be potentially used as biopesticide [48].

### 5.3. Medical Application

Chitinase is used as antifungal agent in combination with antifungal drugs in therapy for various fungal infections [49]. Human AMCase is found to increase in Th2 inflammation and is considered to play a role in asthma and allergic reactions. It is also involved in effector pathway of IL-13 [50]. Chitinase is also being suggested to be used for detection of invasive fungal infection in humans [51]. Chitooligosaccharides also have an enormous pharmaceutical potential to be used in human medicines because of its antitumor activity (shown by chitohexaose and chitoheptaose), wound healing property, and antihypertensive activity [52]. N-Acetyl glucosamine, which is a monomeric unit of chitin polymer, is also reported to be anti-inflammatory agent [53].

*Plasmodium Falciparum* is also reported to produce chitinase enzyme in the sporogonic cycle. Chitinase is produced by pathogen in the midgut of anopheline vector to disrupt the peritrophic membrane and let the parasite get to the salivary glands [54]. Inhibition of chitinase can stop the sporogonic cycle, so it can be considered a good target.* Plasmodium falciparum* forms a membrane sac around the ingested blood meal, that is, chitin containing peritrophic matrix, and thus addition of exogenous chitinases to blood meal prevents the formation of peritrophic matrix [55].

### 5.4. Miscellaneous Applications

Chitinases have been exploited to isolate fungal protoplasts that are used as experimental means to study the synthesis of cell wall, synthesis and secretion of enzymes, and strain improvement for biotechnological applications [56].

The level of chitinases can also be used for the indirect determination of fungal biomass present in the soil. An enzyme of food industry purpose tannase is produced by* Aspergillus niger* but tannase binds to its cell wall reducing the yield. Chitinase is used for fungal cell wall degradation that releases tannase from cell wall and increases the yield [57].

## 6. Future Prospective

**Figure 4:**
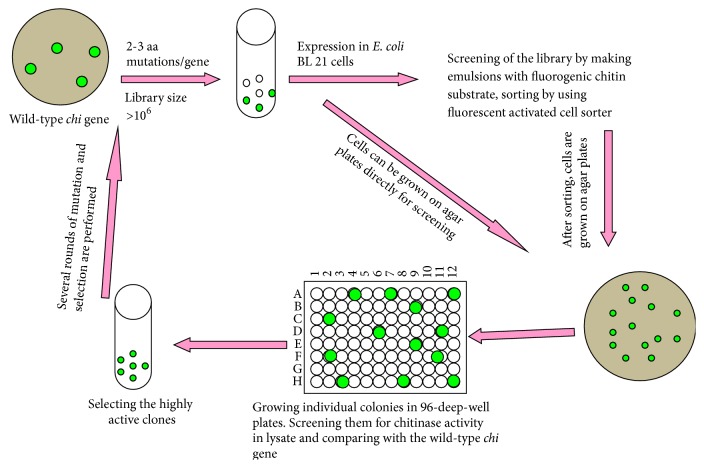
A schematic diagram showing methodology of directed laboratory evolution technique for the creation of new enzymes with enhanced catalytic activity. Chitinase gene library is created by random mutagenic PCR, which randomly incorporates desired number of amino acid mutations. Then the PCR product is cloned, expressed, and screened for higher catalytic activity. For detailed methodology, kindly refer to [9, 10].

Chitinase being the hydrolyzing enzyme for the second most abundant naturally occurring polymer holds many applications in the field of environmental, therapeutic, and industrial biotechnology. Economical and practical utilization of chitinases can be achieved either by high yield or better catalytic activity. Main focus in chitinase research is on improving its catalytic activity. Protein engineering of enzyme is providing a way to achieve this goal. Two major approaches for engineering protein are directed evolution and site directed mutagenesis. In directed evolution approach, chitinase can be randomly mutated by error prone PCR and thereby generation of a mutant library. The chitinase gene library can then be screened by using fluorescent activated cell sorter and/or in 96-well plates. Mutants showing higher activity as compared to wild type can be selected and combination of these positive mutations can also be attempted to get the best catalytic activity (Figure 4) [9, 10].

In case of site directed mutagenesis, specific amino acid residues residing in the chitin binding and catalytic sites are mutated. For instance, glutamate present in the active site of chitinase enzyme plays a major role in catalytic activity by acting as a proton donor for glycosidic bond cleavage [58]. So, any mutation near glutamate that enhances its proton donating efficiency can enhance enzymatic activity. To reduce steric hindrance, bulky group carrying amino acids at the edge of active site could be replaced by small noninteractive amino acid, for example, alanine [59]. Some residues far from the active site also play role in binding of chitinase to the enzyme. Processivity of chitinase can be enhanced by placing hydrophobic amino acid at the edge of active site to make a narrow groove above active site. Due to this narrow groove, chitinase will not release the polymeric chain after every cleavage but will continue on cleaving glycosidic bonds in a processive fashion. Domain swapping is another approach for enhancing chitinase activity. Many chitinases showed low catalytic activity due to lack of chitin binding domain. The chitin binding domain from an active chitinase can be swapped by applying protein engineering methodology. This could increase the catalytic activity by improving substrate binding of the engineered chitinase. The above-mentioned approaches of protein engineering hold huge potential for the improvement of chitinolytic enzymes for future applications.
